# Quantification of Urinary Exosomal Prostate-Specific Antigen for the Diagnosis of Prostate Cancer Using Clinical Laboratory–Based Techniques: Protocol for a Case-Control Study

**DOI:** 10.2196/63551

**Published:** 2024-09-11

**Authors:** Guorong Li, Yannick Tholance, Nora Mallouk, Louis Waeckel, Pascale Flandrin, Bruno Bali, Lionel Badet, Pierre Cornillon

**Affiliations:** 1 Department of Urology North Hospital CHU Saint-Etienne Saint-Etienne France; 2 Laboratory of Biochemistry North Hospital CHU Saint-Etienne Saint-Etienne France; 3 Center of Electronic Microscopy CMES, Faculty of Medicine University of Jean Monnet Saint-Etienne France; 4 Laboratory of Flow Cytometry, Immunology Laboratory North Hospital CHU Saint-Etienne Saint-Etienne France; 5 Laboratory of Molecular Biology North Hospital CHU Saint-Etienne Saint-Etienne France; 6 Department of Medical Oncology North Hospital CHU Saint-Etienne Saint-Etienne France

**Keywords:** liquid biopsy, urinary exosome, diagnosis, PSA, prostate-specific antigen, prostate cancer

## Abstract

**Background:**

Prostate cancer is the most common cancer in men and represents a major public health problem. The current method for the diagnosis or screening of prostate cancer is invasive and costly. There have been renewed and innovative studies searching for urinary biomarkers to aid in the diagnosis of prostate cancer, especially with technologies based on urinary exosomes. However, technologies based on urine exosomes usually need expensive machines such as an ultracentrifuge and they are difficult to standardize, which hinder their application in clinical laboratories. We have optimized and standardized the isolation of urinary exosomes with the precipitation method. We have found that urinary exosomal prostate-specific antigen (PSA) can be quantified by automatic Elecsys total PSA technique.

**Objective:**

In this study, our objective is to utilize urinary exosomes from prostate cancer for the development of a test to aid in its diagnosis.

**Methods:**

Exosomes from the prostate cancer cell line LNCaP was used to set up the technique. To analyze urine samples from patients, the methods include the collection of first-void urine using the Colli-Pee device, the isolation of urine exosomes using the optimized precipitation method, and the quantification of exosomal PSA by Elecsys total PSA.

**Results:**

This will be a 2-year study. We will start including patients and controls in the last quarter of 2024. We expect the results to be published in the second quarter of 2027.

**Conclusions:**

This is the first study to quantify urinary exosomal PSA using the Elecsys total PSA technique for the diagnosis of prostate cancer. This study emphasizes techniques that are suitable for implementation in clinical laboratories, which will facilitate the application of urinary exosomes to simplify and improve the diagnosis and screening of prostate cancer.

**International Registered Report Identifier (IRRID):**

PRR1-10.2196/63551

## Introduction

With 50,000 to 70,000 new cases per year, prostate cancer is the most common cancer in men [1]. The current method for the diagnosis or screening of prostate cancer is based on a biopsy of prostate tissue following an elevated blood concentration of prostate-specific antigen (PSA; >4.0 ng/mL). This method has some drawbacks. Prostate tissue biopsy is invasive and painful with possible side effects for patients. PSA is not a specific marker for prostate cancer. In addition to prostate cancer, other factors can lead to an elevated PSA, including age, prostate infection, and benign prostatic hyperplasia. According to the PSA threshold (usually 4.0 ng/mL), its estimated sensitivity ranges from 67.5% to 80% and its estimated specificity ranges from 60% to 70% [2-4]. Consequently, 20% to 30% of cancers go undetected when only the PSA blood test is considered, and it is not feasible to perform prostate biopsies on a large number of patients. It is more worthwhile to improve the diagnosis and screening of prostate cancer.

Extracellular vesicles are characterized by a lipid bilayer membrane and are subcategorized into small extracellular vesicles (exosomes) and large extracellular vesicles [5]. The size of exosomes is between 30 to 150 nm. Exosomes form within multivesicular bodies and are then released into the extracellular medium by exocytosis [5]. Almost all cell types can secrete exosomes. Exosomes are involved in multiple processes of tumorigenesis and development, including the promotion of angiogenesis, differentiation and infiltration, regulation of immunity, and response to treatment [6,7]. They are widely present in bodily fluids, including blood, saliva, urine, breast milk, pleural fluid, and ascites [6,7]. Since exosomes carry tumor markers such as nucleic acids, proteins, and lipids, they can serve as a liquid biopsy to aid in the diagnosis of tumors.

Urine has the advantage of containing an abundance of exosomes. Because the prostate and urine are in close proximity, prostatic exosomes can be secreted into urine. The characterization of exosomes released by tumor cells and collected in urine could be a new tool to improve the diagnosis of prostate cancer. Recently, we used the exosomes isolated from the prostate cancer cell line LNCaP to set up the techniques. We have optimized and standardized the isolation of urinary exosomes with the precipitation method. We have found that the urinary exosomal PSA can be quantified by automatic Elecsys total PSA technique. Therefore, our objective is to utilize urinary exosomes from prostate cancer for the development of a test to aid in its diagnosis. We hypothesize that urinary exosomal PSA may be a new tool for the diagnosis of prostate cancer.

## Methods

### Overview and Study Design

**Figure 1:**
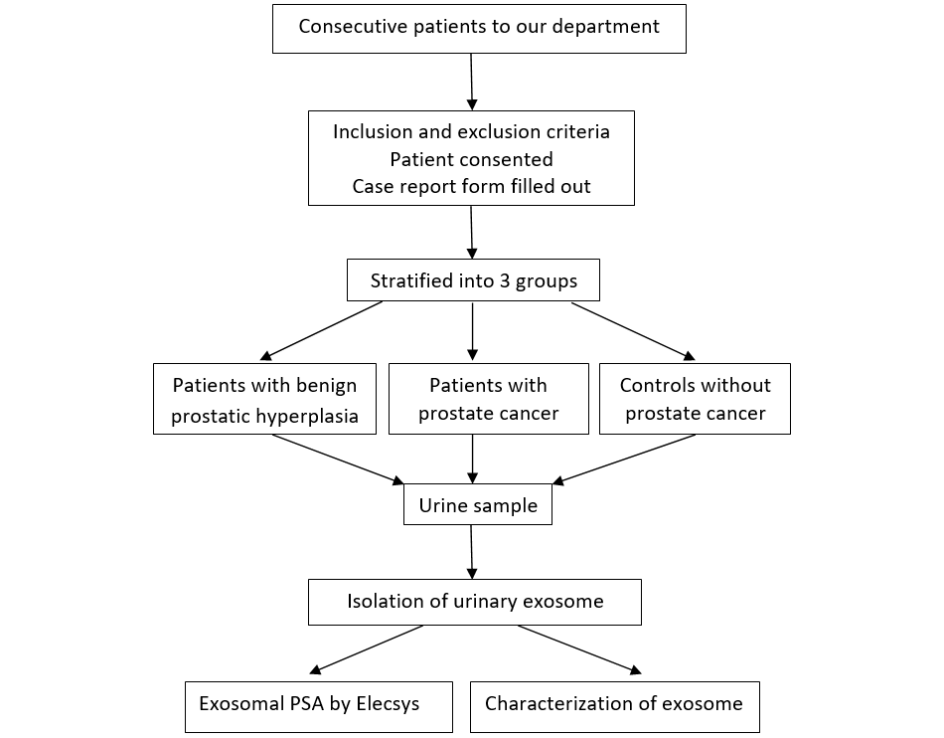
The study scheme. PSA: prostate-specific antigen.

This is a case-control, single-center study. The study team is composed of urologists, oncologists, and biologists from Centre Hospitalier Universitaire (CHU) Saint-Etienne, which is a public and university medical center for patient care and medical research. CHU Saint-Etienne supports medical research projects and provides complete administrative guidance and methodological aid to implement medical research. Before the project began, many meetings were held to discuss the methodological issues as well as administrative issues such as informed consent. Research assistants will be engaged in document collection and sample collection. The study scheme is presented in Figure 1.

### Primary Objective

The primary objective of this study is to quantify urinary exosomal PSA from prostate cancer with clinical laboratory–based techniques.

### Secondary Objective

The secondary objective of this study is to compare the use of transmission electron microscopy (TEM), flow cytometry (FCM), and reverse transcription–quantitative polymerase chain reaction (RT-qPCR) for the detection of tumor urinary exosomes from patients with prostate cancer.

### Primary End Point

The primary end point is to quantify urinary exosomal PSA.

### Secondary End Point

The secondary end point is to compare the percentage of detection according to the different techniques (TEM, FCM, and RT-qPCR).

### Inclusion of Patients With Prostate Cancer

Patients who meet the following criteria are eligible for inclusion into the group of patients with prostate cancer: (1) aged >50 years; (2) a positive biopsy for prostate cancer; (3) without urinary tract infection; (4) benefiting from social security; and (5) having accepted and signed the consent form.

### Inclusion of Patients With Benign Prostatic Hyperplasia

Patients who meet the following criteria are eligible for inclusion into the group of patients with benign prostatic hyperplasia (BPH): (1) aged >50 years; (2) a histologically confirmed diagnosis of BHP; (3) without urinary tract infection; (4) benefiting from social security; and (5) having accepted and signed the consent form.

### Inclusion of Controls

Patients who meet the following criteria are eligible for inclusion into the control group: (1) aged >50 years; (2) normal PSA levels; (3) no clinical evidence of BPH or prostate cancer; (4) prostate volume less than 30 cm^3^; (4) without urinary tract infection; (5) benefiting from social security; and (6) having accepted and signed the consent form.

### Exclusion of Patients and Controls

Patients meeting any of the following criteria are excluded from the study: (1) aged <50 years; (2) taking treatment or hormones known to modify the level of serum PSA in the 3 to 6 months preceding inclusion; (3) a urinary tract infection or history of prostate cancer; (4) history of invasive therapeutic procedures for BPH or for symptoms of urinary tract disorders within 6 months prior to inclusion; (5) a history of concurrent bladder or renal tumors in the 6 months preceding inclusion; and (6) no signature of the consent form.

### Patient Procedure

After checking the inclusion and exclusion criteria, the study information leaflet will be given to patients. Patients will have a 1-hour reflection period. A 20-mL first-void urine sample will be collected using the Colli-Pee device in the department before oncological treatment. Urine collection corresponds to the end of a patient’s participation in this study.

### Urine Sample Processing

Urine samples are kept in refrigerator and processed within 24 hours after collection. Urine is centrifuged at 3000 g for 15 minutes at 4 °C to remove the exfoliated urinary cells. The supernatant is aliquoted and used for the isolation of urinary exosomes.

### Isolation of Urinary Exosomes

To isolate urinary exosomes, 5 mL of the urine supernatant will be used. Urinary exosomes are isolated using the polyethylene-glycol (PEG)–based precipitation method. We have optimized the PEG-based technique for the isolation of urinary exosomes. After the adjustment of urine pH, a solution of PEG is mixed with urine. The mixture is incubated at 4 °C for 2 hours. The mixture is centrifuged at 4500 g at 4 °C to collect urinary exosomes. The exosome pellet is suspended in 200 µL of phosphate buffered saline (PBS) solution for the quantification of PSA.

### Quantification of Urinary Exosomal PSA

In both exosomes isolated from the prostate cancer cell line LNCaP and those isolated from urine samples, the concentration of total PSA is determined in the automatic analyzer Cobas 8000 (Roche Diagnostics). In the Cobas 8000, total PSA is measured in a c602 module using Elecsys total PSA reagent kits based on electrochemiluminiscent immunoassays in a “sandwich” configuration. The concentration of total PSA is expressed in µg/mL.

### Urinary Exosome Characterization

We will characterize urinary exosomes using TEM, FCM, and nanoparticle tracking analysis. TEM is performed on the exosome pellets, which are suspended in PBS. A 10-µL urinary exosome suspension is placed on 200 mesh copper grids (AGS138; Oxford Instruments) and allowed to adhere for 20 minutes. The excess sample is wicked off using a piece of filter paper. The grids are washed 3 times with distilled water. The grids are incubated with a drop of staining solution Uranyless (Delta Microscopy) for 20 seconds and blotted with a filter paper. The urinary exosomes are observed at an accelerating voltage value of 100 kV using a Hitachi electron microscope (H800; Hitachi).

For FCM analysis, the technique uses Aldehyde/sulphate latex beads (A37304; ThermoFisher). First, 1 µL of Aldehyde/sulphate latex beads is added to 100 µL of exosome suspension and mixed well. Second, 900 µL of PBS is then added, and the mixture is incubated for 2 hours at room temperature on a rotary wheel. Third, 100 µL of 100 mM glycine is then added, and the mixture is incubated at room temperature for 30 minutes. The mixture is then pelleted by centrifugation at 2650 g for 5 minutes, and the supernatant is removed. The pellet is washed by the addition of 1mL PBS+0.5% fetal bovine serum solution. The mixture is centrifuged at 2650 g for 5 minutes. Finally, the pellet is resuspended in PBS, and the antibody of antihuman CD63-FITC (IM1165U; Beckman Coulter), CD81-FITC (B25329; Beckman Coulter), or CD9-FITC (IM1755U, Beckman Coulter) or the antibody of isotype control (A07795; Beckman Coulter) is added. The incubation with antibody is performed for 30 minutes in the dark. After twice washing with PBS, the staining is observed by using conventional FCM.

Nanoparticle tracking analysis for urinary exosomes is performed using NanoSight LM10 fitted with a 488-nm laser and 500-nm long-pass filter (Malvern Instruments). The urinary exosome sample is diluted in PBS to a final volume of 1 mL. The diluted sample is then injected into the laser chamber. Particle counts per mL and size distribution of particles in solution are collected and calculated.

### Sample Size

To calculate the necessary number of patients with prostate cancer needed for inclusion, our hypotheses were as follows: 75% detection is considered insufficient (P0=0.75) and 90% is sufficient (P1=0.90), unilateral α=5%, and power=95%. Based on these assumptions, using the 1-step Fleming method, 65 participants are required. To verify the specificity, we will also include 65 patients with BPH and 65 controls.

### Statistical Analysis

For quantitative analysis, the number of observations available, mean, SD, median, minimum, and maximum will be calculated and compared. For qualitative analysis, absolute and relative frequencies (expressed in percentages) will be calculated and compared. Sensitivity and specificity will be calculated. The area under the receiver operating characteristic curve will be determined.

### Data Sources

The sponsor (via the research technicians or the investigators) aims to respond to any request for access to data within a maximum of 1 month. Furthermore, only personnel authorized by the sponsor (investigators, research engineers, and research technicians) and representatives of the health authorities will have access to this information.

Study data will be collected directly in observation notebooks. These data will be validated by the investigator, who will sign (electronically) the observation notebooks.

The information collected as part of this study includes (1) demographic data (age, gender, weight, and height); (2) medical history (stage and grade of cancer); and (3) results of different analysis techniques.

Missing data will need to be justified. Any correction made in the case report form must be traceable.

### Ethical Considerations

This study has received ethics approval by the ethics committee of CHU Saint-Etienne (IRBN262024/CHUSTE). No compensation will be provided to the participants.

## Results

This will be a 2-year study. We will start including patients and controls in the last quarter of 2024. We expect the results to be published in the second quarter of 2027.

## Discussion

The study of urinary markers for the diagnosis of prostate cancer is a renewed and innovative research area in the contemporary era [8,9]. Liquid biopsy using urinary exosomes is promising for the diagnosis of prostate cancer. According to recent studies, tumor can discharge more exosomes into bodily fluids. We expect the quantity of urinary exosomes from patients with prostate cancer to be significantly increased compared to that of controls. Therefore, the exosomal PSA level would be significantly increased in patients with prostate cancer. The increased exosomal PSA level may be a new tool for the diagnosis of prostate cancer. A recent study supports that the level of urinary exosomal PSA is increased compared to that of patients with BPH or normal controls [10]. However, to implement the urinary exosomal PSA testing into clinical laboratories, improvement or optimization is necessary for every step, such as urine collection, the isolation of urinary exosomes, and the quantification of urinary exosomal PSA. This study is the first one to optimize and standardize the precipitation method for isolating urinary exosomes.

This study presents some unique characteristics: the collection of first-void urine using a standardized Colli-Pee device, the isolation of urine exosomes using an optimized precipitation method, and the quantification of exosomal PSA by Elecsys total PSA. First-void urine is important because it contain more exosomes from the prostate. In this study, first-void urine is to be collected without digital prostate message, whereas a previously published study required a digital prostate message before the collection of urine [10]. At present, there has been no consensus on the isolation method. Methods that are suitable for clinical laboratories are preferable. In this study, we decided to adapt the precipitation method. The precipitation technique using PEG for the isolation of exosomes is considered suitable for clinical laboratories [11]. Many commercial kits such as the commercial ExoQuick kit use this technology. We have optimized and standardized the precipitation method to isolate urinary exosomes. We recently found that urinary exosomal PSA could be detected by the Elecsys total PSA automatic assay in a clinical laboratory. To the best of our knowledge, it is the first study to detect urinary exosomal PSA from prostate cancer using the Elecsys total PSA technique.

Urinary exosomes from prostate cancer express many tissue markers. The contents in urinary exosomes are relatively stable because of the protection by a lipid bilayer membrane. Many tumor markers including PSA, prostate-specific membrane antigen, and prostate cancer antigen 3 have been identified in urinary exosomes as being of potential interest [12-14]. Recent results show that urinary exosomes are particularly useful for those who need a prostate biopsy, that is, men with a PSA level between 2-10 ng/mL [15,16]. By avoiding unnecessary tissue biopsy, it is considered as a major advancement in the diagnosis of prostate cancer [15,16]. To characterize urinary exosomes from prostate cancer, we will use the RT-qPCR, FCM, and TEM techniques to study the exosomal markers. Conventional FCM seems to not be suitable for the detection of exosomes because of the size detection limit. For FCM, we will use the bead-assisted technique [17,18] and the classic technique of detection by FCM on a DXFLEX cytometer. The bead-assisted technique is interesting. The RT-qPCR technique is routine in clinical laboratories. We will perform RT-qPCR according to the TaqMan technique [17,18]. We have worked on cancerous exosomes in biological fluids for several years [17-22]. Recently, we have developed an immunostaining and TEM observation test to identify exosomes from clear cell kidney cancer [19]. We wish to use this technique to characterize tumor exosomes in the urine of patients with prostate cancer. In this project, we will use immunolabeling of exosomal and tumor biomarkers and observation of exosomes under TEM to further confirm our objective.

In conclusion, this is the first study to quantify urinary exosomal PSA using the Elecsys total PSA technique for the diagnosis of prostate cancer. This study emphasizes techniques that are suitable for implementation in clinical laboratories, which will facilitate the application of urinary exosomes to simplify and improve the diagnosis and screening of prostate cancer.

## Abbreviations

BPH: benign prostatic hyperplasia
CHU: Centre Hospitalier Universitaire
FCM: flow cytometry
PBS: phosphate buffered saline
PEG: polyethylene-glycol
PSA: prostate-specific antigen
RT-qPCR: reverse transcription–quantitative polymerase chain reaction
TEM: transmission electron microscopy

## Data Availability

The datasets generated and analyzed during this study are not publicly available to maintain participant confidentiality but will be available from the corresponding author upon reasonable request.
